# Supplementary material for: Validation of the StimQ2: A parent-report measure of cognitive stimulation in the home
Source: PLoS One. 2023 Jul 24;18(7):e0286708. doi: 10.1371/journal.pone.0286708 (PMC10365315; doi:10.1371/journal.pone.0286708)
Supplement: S1 Appendix — (PDF) [file pone.0286708.s001.pdf]

S1 Appendix. Possible score ranges for the StimQ2

**Infant**	Possible Range
**READ Subscale**	**0 – 15**
Bookreading Quantity	0 – 9
Diversity of Bookreading Concepts/Content	0 – 2
Bookreading Quality	0 – 4
**PIDA Subscale**	**0 – 5**
**PVR Subscale**	**0 – 16**
Parental Verbal Responsivity During Everyday Routines	0 – 9
Parental Verbal Responsivity During Play and Pretend	0 – 7
**TOTAL Core Score [READ + PIDA + PVR]**	**0 – 36**
**ALM Subscale**	**0 – 6**
First Infant Toys	0 – 2
Activity/Manipulative Toys	0 – 2
Imagination Toys	0 – 2
**TOTAL Score [READ + PIDA + PVR + ALM]**	**0 – 42**

**Toddler**	Possible Range
**READ Subscale**	**0 – 19**
Bookreading Quantity	0 – 9
Diversity of Bookreading Concepts/Content	0 – 6
Bookreading Quality	0 – 4
**PIDA Subscale**	**0 – 5**
**PVR Subscale**	**0 – 15**
Parental Verbal Responsivity During Everyday Routines	0 – 9
Parental Verbal Responsivity During Play and Pretend	0 – 6
**TOTAL Core Score [READ + PIDA + PVR]**	**0 – 39**
**ALM Subscale**	**0 – 7**
Symbolic Play	0 – 2
Art	0 – 1
Adaptive/Fine Motor	0 – 2
Language	0 – 1
Life-size	0 – 1
**TOTAL Score [READ + PIDA + PVR + ALM]**	**0 – 46**

**Preschool**	Possible Range
**READ Subscale**	**0 – 18**
Bookreading Quantity	0 – 9
Diversity of Bookreading Concepts	0 – 2
Diversity of Bookreading Content	0 – 2
Bookreading Quality	0 – 5
**PIDA Subscale**	**0 – 15**
PIDA of Emergent Literacy	0 – 7
PIDA of Math and Spatial Orientation	0 – 8
**PVR Subscale**	**0 – 19**
Parental Verbal Responsivity During Everyday Routines	0 – 9
Parental Verbal Responsivity During Play, Pretend, Imagination	0 – 6
Parental Verbal Responsivity During Activities that Promote Regulation	0 – 4
**TOTAL Core Score**	**0 – 52**
**ALM Subscale**	**0 – 8**
Symbolic Play	0 – 2
Art	0 – 2
Adaptive/Fine Motor	0 – 2
Language/Concepts	0 – 2
**TOTAL Score [READ + PIDA + PVR + ALM]**	**0 – 60**
