# Supplementary material for: Validation of the StimQ2: A parent-report measure of cognitive stimulation in the home
Source: PLoS One. 2023 Jul 24;18(7):e0286708. doi: 10.1371/journal.pone.0286708 (PMC10365315; doi:10.1371/journal.pone.0286708)
Supplement: S1 Table — (PDF) [file pone.0286708.s002.pdf]

S2 Table. Number of items comprising each subscale of the original StimQ and $StimQ_2$

	Original StimQ	Added	Dropped	$StimQ_2$
**Infant**	52	14	9	57
ALM	22	3	2	23
READ	12	5	3	14
PIDA	7	0	2	5
PVR	11	6	2	15
**Toddler**				
ALM	39	0	2	37
READ	11	4	1	14
PIDA	10	0	5	5
PVR	4	10	0	14
**Preschool**				
ALM	40	0	9	31
READ	15	6	4	17
PIDA	15	2	2	15
PVR	7	12	5	14
Note: PVR = Verbal Responsivity, READ = Reading, PIDA = Teaching, ALM = Learning Materials				
